# The gut microbiota in menopause: Is there a role for prebiotic and probiotic solutions?

**DOI:** 10.1177/20533691251340491

**Published:** 2025-05-07

**Authors:** Marrium Liaquat, Anne Marie Minihane, David Vauzour, Matthew G Pontifex

**Affiliations:** 1 Norwich Medical School, 6106 University of East Anglia, Norwich, UK

**Keywords:** Menopause, diet, microbiome, estrogen, fibre

## Abstract

The gut microbiota, comprising a diverse array of microorganisms in the gastrointestinal tract, has emerged as a key player in human health. Emerging research indicates that this gut microbial composition is influenced by sex. These sex differences are not necessarily static and likely alter across the life course in response to several factors including changing hormone profile. As such, the menopause transition-a pivotal phase in female ageing in which the hormone profile changes dramatically is receiving increasing attention. Declining estrogen which occurs during menopause appears to influence the microbiota, which may in turn contribute to menopause-related conditions such as weight gain, bone health, cancer risk and cognitive health. The modulation of estrogen through the gut’s ‘estrobolome’, a collection of bacterial genes involved in estrogen metabolism, may offer explanation for some of the interindividual differences observed during menopause (e.g. length, symptoms and disease risk). Therapeutic modulation of the gut microbiota therefore represents a potential approach towards managing menopausal symptoms. Indeed, prebiotics and probiotics such as *Lactobacillus* have been shown to increase bacterial diversity and improve metabolic and overall health in menopausal women. However, evidence remains limited regarding the specific underlying mechanisms, highlighting an urgent need for a research focus in the area. This review summarizes the current understanding of the gut microbiota’s role in menopausal health and the potential of prebiotics and probiotics as therapeutic interventions. Further research into gut microbiota modulation may enable more effective, personalised treatments for menopause-associated health challenges, and supporting women’s health into older ages.

## Introduction

The ‘Gut microbiota’ refers to the collection of microorganisms in the gastrointestinal (GI) tract including bacteria, archaea, viruses and fungi.^1^ A symbiotic relationship exists between these organisms and the host, which can be beneficial, neutral or pathogenic. The gut microbiota is shaped and influenced by various factors (exogenous and endogenous) encountered throughout the life course including but not limited to birth (e.g. mother’s health and mode of delivery), diet and antibiotic usage. Intriguingly, biological sex has also been identified as an important modulator of gut microbial composition.^1^ Sex mediated gut microbiota differentiation starts in early life. On the first day of life, male infants show higher *Bifidobacterium* levels as compared to female infants.^2^ Within the first 30 days, stool sample analysis reveals that male neonates have lower α-diversity, lower abundance of *Clostridiales* and higher abundance of *Enterobacteriales* compared to females.^3^

Both dynamic changes (during the onset of puberty and menopause) and steady-state (during adulthood) levels of sex hormones play a role in shaping the gut microbiota.^1^ In adulthood, gut microbial diversity continues to increase and transitions towards a state of uniqueness (within each person) with advancing age,^4^ plateauing typically around the age of 40 years.^5^ Sexual dimorphism of the gut microbiota, in adulthood, has been observed in several human studies.^6–9^ In two large human cohorts, the Belgian Flemish Gut Flora Project (*n* = 1106) and the Dutch LifeLines-Deep study (*n* = 1135), sex (10th strongest effect size among 69 factors) correlated significantly with overall microbiome community variation.^9^ Another large cohort study of adults from four geographical regions showed that young adult women had higher gut microbial diversity than young adult men, but these differences were less pronounced in middle-aged adults.^5^ Differences in gut microbiota between pre- and post-menopausal women have been reported,^10^ with postmenopausal women’s gut microbiota observed to be more like age-matched men than premenopausal women.^11^ A higher relative abundance of *Lachnospira* and *Roseburia*, and a lower relative abundance of *Prevotella*, *Parabacteroides* and *Bilophila* were reported in premenopausal women than in postmenopausal women (who had similar levels to the men).^11^

Although research on gut microbiota changes during the menopausal transition is emerging. The available studies are inconsistent, with most of them comparing menopausal women with non-menopausal women, which does not account for the age effect. Thus, the current evidence remains limited, highlighting the need for further investigation. This review aims to summarise the existing knowledge on the interplay between the gut microbiota and sex hormones and their potential role in menopausal symptoms. We also present the current evidence on the impact of prebiotic and probiotic interventions (potent gut microbial modulators) and their role in alleviating menopausal symptoms.

## Gut microbiota and sex hormones

Sex steroid hormones (including androgens, estrogens and progestogens) are the main determinants of metabolic and physical differences between females and males. Generally, females and males have the same hormones but they differ in their blood concentrations, production sites, and their interaction with different organs and systems.^12^

A bidirectional relationship exists between these sex hormones and the gut microbiota: (1) sex hormones (at specific concentrations) appear to modulate the gut microbial composition,^13^ whilst (2) the gut microbiota regulates the levels of circulating sex hormones.^14^

2.1) Rodent studies have been particularly useful in establishing the modulatory effects of sex hormones on the microbiota. For example, gut bacterial diversity is observed to be similar across males and females prior to puberty, with differences only becoming clear among post-pubescent animals.^15^ Similarly, castrated male littermates possess a microbiota more like that of a female microbiota.^15^ This sex-specific difference in microbiota was found to be consistent across different strains of mice.^16^

In humans this is more difficult, observations in pregnant women have revealed profound effects on the gut microbiota, especially during the third trimester when the estrogen levels are at their peak.^17^ Menopause associated sex hormones changes are also linked with decreased gut microbial diversity in women as the microbiota shifts towards more male-like composition.^14^ More research needs to be done to better understand these changes, and their role in menopause-linked health status.

**Figure 1.**
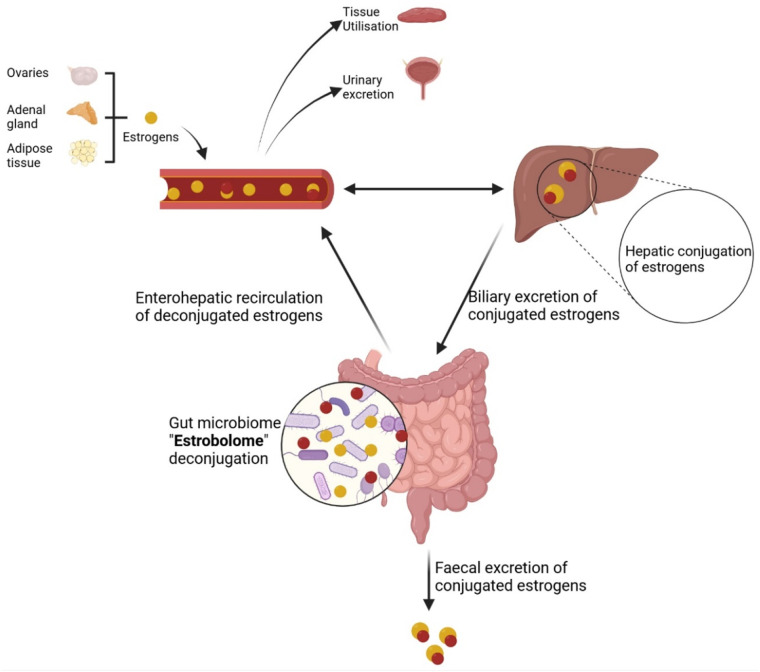
Estrogens are produced by the ovaries, adrenal gland and adipose tissue, and released in the blood stream, and metabolised (in the liver) into biologically inactive conjugated forms which are released via biliary excretion into the small intestine. A specific set of gut bacteria, possessing β-glucuronidase enzymes (referred to as ‘Estrobolome’) deconjugate estrogens into biologically active free-forms. These may be reabsorbed into the blood circulation, used by peripheral tissues, and returned to the liver through enterohepatic recycling for re-conjugation. Some of the conjugated and deconjugated estrogens are excreted via urine and stool.

2.2) Endogenous estrogens are metabolised in the liver into glucuronidated or sulphated forms, which allows the conjugated estrogens to reach the intestinal tract via biliary excretion. A number of gut bacterial species across major phyla, and especially Firmicutes and Bacteroidetes, possess enzymes like β-glucuronidases^18,19^ and sulphatases,^20^ which deconjugate the excreted estrogens, thus allowing them to be recycled back into the circulation and carry on their physiological function^21^ (Figure 1). An aggregate of these bacterial genes, whose products are capable of metabolising estrogens, is referred to as the ‘estrobolome’.^22^ This recycling was further confirmed using radioactively labelled estradiol and estrone when injected in women, and their excretion was recorded in bile and faeces. Approximately, 50% of the injected estrogens are excreted in bile,^23^ with a much smaller fraction appearing in the faeces (10–15%)^24^ which indicates that a significant portion is reabsorbed in the systemic circulation via bacterial deconjugation. Other sex hormones including progesterone^25^ and androgens^26^ are also similarly deconjugated and recirculated by the gut microbiota. In a recent human study, the estrogen metabolising activity of the gut microbiota (β-glucuronidase enzyme activity) was found to be inversely associated with estrogen levels in the gut, whereas the systemic levels of estrogens were strongly and directly associated with gut microbial richness (i.e. number of unique bacterial species).^27^ The role of gut microbiota in modulating sex hormones is further supported by a rodent study in which the transplantation of gut microbiota from male to female mice resulted in a systemic increase in testosterone levels.^28^

## Menopausal transition, gastrointestinal health and the gut microbiota

**Figure 2.**
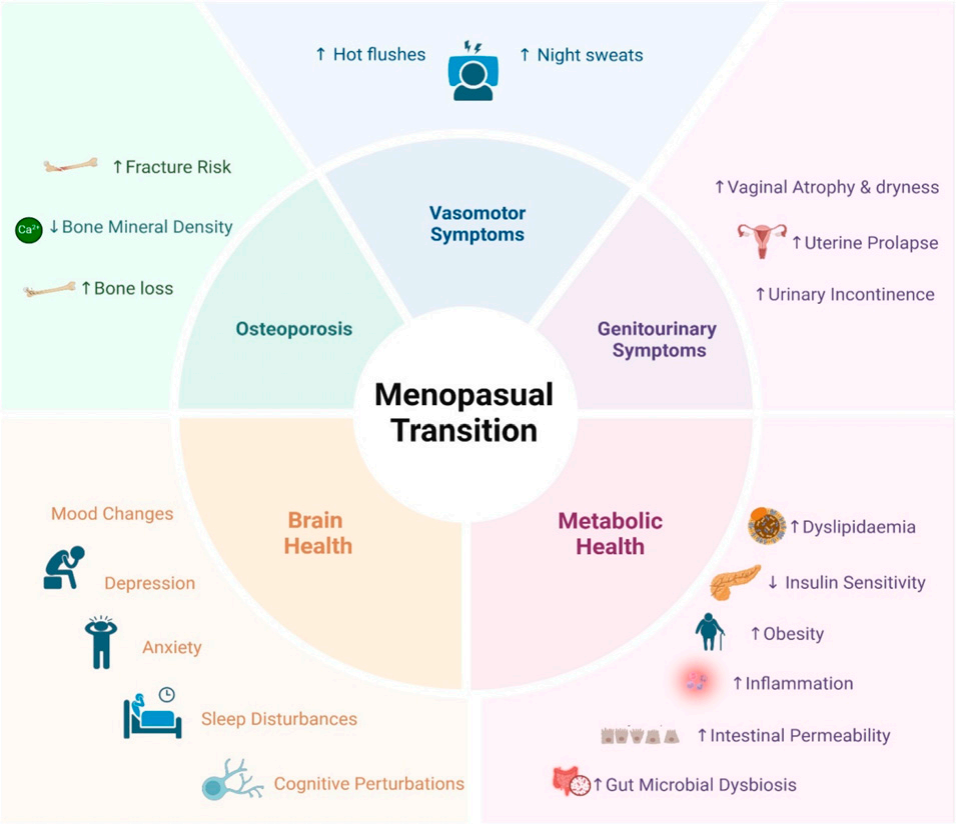
Representation of physiological symptoms associated with menopausal transition.

The menopausal transition occurs as a result of natural reproductive ageing when a woman loses primary ovarian follicles and oocytes. It is accompanied by fluctuations in female sex hormones including a decrease in estrogens and progesterone; and an increase in follicle-stimulating hormone (FSH) and luteinising hormone (LH).^29^ These hormonal fluctuations are accompanied by various menopausal symptoms (varying in severity among women) including vasomotor symptoms (i.e. hot flushes and night sweats), genitourinary symptoms (i.e. vaginal atrophy and dryness, and urinary incontinence), mood changes (i.e. depression and anxiety), cognitive perturbations (termed brain fog), sleep disturbances and changes in bone mineral density (Figure 2).^30^ The menopausal transition usually lasts for an average of 7 years and occurs between the ages of 45 and 55 years.^29^ Delayed menopause is associated with higher risk of endometrial and breast cancer, while early menopause increases the risk for osteoporosis and cardiovascular diseases. The age of natural menopause onset depends on various factors including genetic, environmental, reproductive, dietary and lifestyle.^31^ The association between diet and menopause onset is not very well researched but there is evidence that dietary factors influence the age of menopause onset via their effect on serum hormone levels.^31,32^

Menopause significantly impacts gastrointestinal health, influencing appetite regulation, weight management and digestion, all of which are linked to hormonal changes during this stage.^33^ Postmenopausal women experience changes in bowel function more often compared to premenopausal women,^34^ with gastrointestinal conditions such as irritable bowel syndrome presenting more severe symptoms in postmenopausal women than in premenopausal women or men of similar age.^35^ Hormonal shifts associated with menopause commonly lead to these gastrointestinal symptoms including abdominal pain, bloating, nausea, constipation and indigestion,^36^ which may also be associated with alterations in the gut microbial composition.

As aforementioned, the gut microbiota plays an important role in maintaining circulating levels of sex hormones. Experimental evidence suggests that estrogen and progesterone improve barrier function of intestinal epithelial cells by upregulating the tight junction proteins.^37,38^ The hormonal fluctuations and decline associated with menopause have been linked to reduced gut barrier integrity and increased microbial translocation.^14^ Similarly, in mouse model, ovariectomy has been shown to increase intestinal permeability.^39^ Findings from the Study of Women’s Health Across the Nation (SWAN) cohort indicate that gut permeability (as measured by fatty acid binding protein 2 [FABP2]), increases during the menopausal transition and is linked to heightened inflammation.^40^ This increased permeability facilitates the movement of microbes and microbial products from the gut into systemic circulation, potentially triggering systemic inflammation and immune activation, which are associated with the pathogenesis of various diseases.^41^ Menopause affects the gastrointestinal health as well as gut microbiota composition which potentially play an important role in menopause-associated symptoms.

A metagenome-wide association study identified distinct differences in the gut microbiota and associated metabolites between premenopausal and postmenopausal women.^10^ Postmenopausal women exhibited an overrepresentation of *Bacteroidetes* and *Tolumonas*, while *Firmicutes* and *Roseburia* species were depleted.^10^ *Roseburia,* a group of commensal bacteria that produce short-chain fatty acids, are crucial for metabolic health, and their reduction has been linked to various metabolic diseases.^42^ Conversely, *Tolumonas* showed a negative correlation with bone mineral density,^10^ potentially due to its role in toluene production, which adversely affects bones by reducing bone density.^43^ In addition to differences in bacterial species, significant variations in the metabolic and biological processes of these species were observed between premenopausal and postmenopausal women through GMM analysis. These findings suggest a potential contribution to increased disease risks in postmenopausal women..^10^ Another study comparing gut microbiota between individuals with menopausal syndrome (MPS) and healthy menopausal subjects revealed that gut dysbiosis was present exclusively in those with MPS. MPS is diagnosed by clinical manifestations including (1) menstrual disorders; (2) vasomotor symptoms primarily hot flushes; (3) one or more additional symptoms such as a mental disorder, urogenital atrophy, cardiovascular symptoms, skin and body changes and osteoporosis; along with (4) menopause-related hormonal changes.^44^ These individuals exhibited an enrichment of metabolic pathways associated with cardiovascular disease and carbohydrate metabolism, suggesting a higher risk of developing cardiovascular disease, obesity, diabetes, and other related conditions.^44^

Notably, menopause-associated gut microbial dysbiosis has been linked to postmenopausal osteoporosis. Changes in bacterial α-diversity (the number and distribution of unique bacterial species) and β-diversity (patterns of microbial shifts) were more pronounced in postmenopausal osteoporosis patients compared to non-osteoporotic postmenopausal women. These microbial alterations were also associated with changes in bone mineral density.^45^ In postmenopausal obese women, visceral adiposity was positively associated with gut dysbiosis, and negatively associated with the *Firmicutes:Bacteroidetes* ratio. It was observed that visceral adiposity in these postmenopausal women was also inversely associated with the abundance of SCFA-producing commensal bacteria.^46^ Taken together, literature suggests the association of gut dysbiosis with not only menopausal symptoms but also the severity of these symptoms, although further research is needed to better understand the underlying mechanisms.

## Gut-microbiota-brain axis in menopause

Menopause affects brain health altering connectivity, structure and metabolism independent of age.^47^ Indeed, brain fog and issues with memory are often reported during the menopause transition.^48^ Sex hormones such as estrogen have implications for brain health with estrogen described as neuroprotective, promoting spinogenesis and synaptogenesis,^49^ as well as cerebral blood perfusion and overall cerebrovascular function.^48^ As such, it is possible that the modulation of estrogens through the ‘estrobolome’ could have neurological implications. Similarly, it could be envisaged that remodelling of the microbiome through various interventions (e.g. dietary interventions) could improve/restore gut brain axis interactions impacted by menopause. To our knowledge a gut-microbiota-brain axis connection in relation to menopause is yet to be reported but should be the focus of future research endeavours given the clear impact of menopause on brain health.

## Menopausal transition – prebiotic and probiotic interventions and future directions in microbiota research

Hormone Replacement Therapy (HRT) is often the first line of treatment for improving menopausal symptoms. However, its use is contraindicated for many women, for example, those with a history of breast cancer or blood clotting, with many women also suffering side effects and not wishing to take HRT over extended periods of time.^30^ Therefore, alternatives to HRT in menopause are needed.

Modulating the gut microbiota might be a promising strategy to alleviate or relieve menopausal symptoms.^49^ The gut microbiota primarily feed on dietary fibres. High quality carbohydrate intake (which means higher intake of fibres, solid carbohydrates and low glycaemic index foods) has been associated with lower somatic and psychological symptoms of menopause.^50^ Prebiotic interventions have shown promising potential in managing menopausal symptoms by modulating the gut microbiota and addressing associated physiological and metabolic changes (Table 1). Dietary interventions with prebiotics such as flaxseeds^51^ and vegetables^52^ have improved intestinal integrity, lipid profiles, and been associated with reductions in body weight and BMI among perimenopausal and menopausal women. Similarly, flavonoid-rich blackcurrants^53^ and water-soluble soybean fibres^54^ have shown benefits for bone health by enhancing calcium absorption and preserving bone mineral density, thereby mitigating osteoporosis risks in menopausal women. Metabolic health improvements have also been observed with flaxseed mucilage resulting in better insulin sensitivity^55^ in obese postmenopausal women. Additionally, rice bran and tea seed oil have reduced both peripheral and neuroinflammation in animal models,^56^ while prebiotic-rich yogurt alleviated menopausal symptoms such as anxiety, depression and vasomotor disturbances.^57^

**Table 1.** Studies on the effect of prebiotics in managing menopausal symptoms.

Study reference	Study population	Study design	Intervention	Study duration	Results/Observed outcomes
Rodent studies					
Lucas et al., 2000^62^	Ovariectomised (OVX) Sprague-Dawley rats (*n* = 48)	Experimental intervention versus sham-operated and OVX controls	Low-dose (LD) prune diet (5%), High-dose (HD) prune diet (25%) prunes	45 days	HD prune diet prevented the OVX induced elevation in serum cholesterol
Mitmura et al., 2003^54^	OVX Sprague-Dawley rats (*n* = 40)	Experimental intervention versus sham-operated and OVX controls	Water-soluble soybean fibre (WSSF) (50 g/kg of diet)	4 weeks	WSSF-fed rats had ↑Ca absorption, ↑femoral Ca content, and prevented the OVX induced elevation in serum cholesterol
Chao et al., 2024^56^	OVX C57BL/6 female mice under multiple metabolic stressors (*n* = 16)	Experimental intervention versus sham-operated young and old mice	Rice bran and tea seed oil (10% *w*/*w* of the diet)	8 weeks	↑ *Clostridia* (short chain fatty acid producers) ↓*Tannerellaceae* (endotoxins producers) ↓hepatic fat accumulation, ↓peripheral inflammation ↓oxidative damage ↓neuroinflammation in the brain
Human studies					
Sari et al., 2020^52^	Menopausal overweight women with hyperlipidaemia (*n* = 60)	Randomised parallel control study	400 g/day of vegetables	21 days	Improvements in plasma lipid profile, body weight, and BMI were observed
Sant’Ana et al., 2022^51^	Perimenopausal overweight women (*n* = 30)	Non-randomised, prospective, parallel clinical trial	Brown and golden flaxseeds (40g)	12 weeks	Flaxseeds consumption ↓intestinal permeability and improved the blood lipid profile, especially in the golden flaxseeds group
Brahe et al., 2015^55^	Obese postmenopausal women (*n* = 58)	Randomised, blinded, placebo-controlled trial	Flaxseed mucilage (10 g/day)	6 weeks	↓serum C-peptide ↓insulin release during an oral glucose tolerance test ↑insulin sensitivity ↓*Faecalibacterium* species Observed changes in insulin sensitivity were not mediated by bacterial changes
Nosal et al., 2024^53^	Peri- and early postmenopausal women aged 45–60 years (*n* = 40)	Randomised, double-blind, placebo-controlled trial	Blackcurrant (BC) supplementation	6 months	- Preserved whole-body bone mineral density - Dose-dependent increase in *Ruminococcus 2* bacteria*,* suggestive of the BC’s bone protective effect
Kahleova et al., 2023^61^	Postmenopausal women (*n* = 84)	Randomised controlled trial	Low-fat, vegan diet with cooked soybeans (1/2 cup daily)	12 weeks	- ↓severe day hot flushes associated with changes in *Porphyromonas* and *Prevotella corporis* abundance -↓total severe hot flushes and ↓severe night hot flushes associated with changes in *Clostridium asparagiforme* abundance
Wu et al., 2022^63^	Postmenopausal women with a history of Roux-en-Y gastric bypass (RYGB) (*n* = 20)	Randomised double-blind, placebo-controlled trial	Soluble corn fibre (SCF) (20 g/day) – a prebiotic	2 months	↑fractional calcium absorption following SCF treatment only in those with ↑change in microbial composition
Shafie et al., 2022^57^	Menopausal women (*n* = 60)	Triple-blind, randomised controlled trial	Prebiotic-rich yogurt (100g)	6 weeks	↓mean total scores of menopausal symptoms ↓anxiety ↓depression ↓vasomotor symptoms

Some individual probiotics and probiotic blends also demonstrate beneficial effects in managing menopausal symptoms (Table 2). *Lactobacillus* strains have shown improvements in managing menopausal symptoms (Table 2). *L. acidophilus* supplementation improved bone mineral density whilst also enhancing trabecular and cortical bone microarchitecture.^58^ *L. intestinalis* YT2 supplementation helped recover OVX-induced gut dysbiosis and improved other symptoms like pain sensitivity, depressive behaviour, fat deposition and bone loss.^49^ *L. gasseri* CP2305 supplementation improved total, vasomotor, somatic and psychological scores in premenopausal women.^59^ A combination of *L. rhamnosus* GR-1 and *L. reuteri* RC-14 improved vaginal flora and overall urogenital health in postmenopausal women.^60^

**Table 2.** Studies on the effect of probiotics supplementation in managing menopausal symptoms.

Study reference	Study population	Study design	Intervention	Study duration	Results/Observed outcomes
Rodent studies					
Dar et al., 2018^58^	Ovariectomised (OVX) mouse model (8–10 weeks old) (*n* = 30)	Experimental intervention versus sham-operated and OVX controls	*Lactobacillus acidophilus* (10^9^ CFU/ml) – a probiotic	6 weeks	↑bone mineral density ↑bone heterogeneity - Enhanced trabecular and cortical bone microarchitecture
Lim et al., 2021^49^	OVX rats (*n* = 60)	Experimental intervention versus sham-operated and OVX controls	*Lactobacillus intestinalis* YT2 (10^9^ CFU/ml) – a probiotic strain	18 weeks	- Recovery of OVX induced gut dysbiosis - Improved menopausal symptoms ↓fat mass ↓pain sensitivity ↓depressive behaviour ↓cognitive impairment ↓bone loss
Human studies					
Honda et al., 2024^64^	Healthy peri- and postmenopausal women (*n* = 111)	Randomised double-blind, placebo-controlled trial	*Levilactobacillus brevis* KABP052 (a probiotic)- as part of a blend containing ≥1 billion CFU	12 weeks	↑estradiol and ↑estrone levels were observed in probiotic group versus placebo
Sawada et al., 2022^59^	Premenopausal women aged 40–60 years (*n* = 80)	Double-blind, placebo-controlled, parallel-group trial	*Lactobacillus gasseri* CP2305 (1 x 10^10^ CP2305 bacterial cells)	6 consecutive menstrual cycles	-Relief in total, vasomotor and psychological score on the Simplified Menopausal Index - Relief in total, somatic, and vasomotor score on Greene Climacteric Scale
Szulinska et al., 2018^65^	Obese postmenopausal women (*n* = 81)	Randomised, placebo-controlled, clinical trial	Ecologic barrier (live multispecies probiotic supplement, 1-2.5 x 10^10^ CFU per day)	12 weeks	↓systolic blood pressure ↓vascular endothelial growth factor ↓serum interleukin-6 ↓serum tumour necrosis factor alpha ↑vascular function
Petricevic et al., 2008^60^	Postmenopausal women with Nugent scores between 4 and 6 in initial vaginal swab (*n* = 72)	Randomised, double-blind, placebo-controlled study	*Lactobacillus rhamnosus* GR-1 and *Lactobacillus reuteri* RC-14 (2.5 x 10^9^ CFU of each strain)	14 days	-Improved vaginal flora -Improved overall urogenital health

While some of these studies have examined gut microbial changes as potential mechanistic drivers,^53,55,56,61^ a significant gap in existing research lies in the inconsistent investigation of these microbial shifts as the underlying mechanism for the observed benefits. Further research into gut microbial changes in response to prebiotic supplementation in menopausal women is essential to deepen our understanding of its role in managing menopausal symptoms. This will not only allow for the development of more targeted and effective therapeutic interventions for menopausal symptom management but could also help identify specific probiotic strains or optimal combinations (e.g. symbiotics) that offer the greatest therapeutic potential.

## Conclusion

In conclusion, while emerging evidence points to the gut microbiota’s role in modulating hormonal balance and potentially influencing menopausal symptoms, there remains a substantial need for focused research to clarify these mechanisms and to determine effective gut microbiota-based interventions. Prebiotics and probiotics offer promising, non-hormonal therapeutic avenues to alleviate menopausal symptoms such as hot flushes, inflammation and metabolic symptoms; however, their role in modulating microbial diversity and microbiota-linked relief in menopausal symptoms needs to be further explored. Most existing studies are primarily observational, focussing on associations rather than establishing causality or elucidating underlying mechanisms. Additionally, many studies compare menopausal women to non-menopausal women without adequately accounting for the effects of age. Future research should address these limitations by incorporating designs that distinguish between age-related and menopause-specific changes, while also focussing on demonstrating causative relationships and mechanisms.

Furthermore, studies should aim to identify specific bacterial strains and prebiotic compounds that are most effective in supporting hormonal health, mood, and metabolic function in menopausal women. Such research could lead to innovative, personalised therapies that not only address the symptoms of menopause but also enhance life-long health and well-being in women. As the field advances, these insights may pave the way for gut microbiota-focused approaches as integral components of menopause management.
